# Gradual Failure of a Rainfall-Induced Creep-Type Landslide and an Application of Improved Integrated Monitoring System: A Case Study

**DOI:** 10.3390/s24227409

**Published:** 2024-11-20

**Authors:** Jun Guo, Fanxing Meng, Jingwei Guo

**Affiliations:** 1 Hunan Institute of Geophysics and Geochemistry, Changsha 410014, China; 2 Hunan Institute of Nuclear Geological Survey, Changsha 410011, China; qinym_dzy@163.com

**Keywords:** rainfall-induced landslide, field exploration, failure mechanism, LoRa Internet of Things, monitoring

## Abstract

Landslides cause severe damage to life and property with a wide-ranging impact. Infiltration of rainfall is one of the significant factors leading to landslides. This paper reports on a phase creep landslide caused by long-term rainfall infiltration. A detailed geological survey of the landslide was conducted, and the deformation development pattern and mechanism of the landslide were analyzed in conjunction with climatic characteristics. Furthermore, reinforcement measures specific to the landslide area were proposed. To monitor the stability of the reinforced slope, a Beidou intelligent monitoring and warning system suitable for remote mountainous areas was developed. The system utilizes LoRa Internet of Things (IoT) technology to connect various monitoring components, integrating surface displacement, deep deformation, structural internal forces, and rainfall monitoring devices into a local IoT network. A data processing unit was established on site to achieve preliminary processing and automatic handling of monitoring data. The monitoring results indicate that the reinforced slope has generally stabilized, and the improved intelligent monitoring system has been able to continuously and accurately reflect the real-time working conditions of the slope. Over the two-year monitoring period, 13 early warnings were issued, with more than 90% of the warnings accurately corresponding to actual conditions, significantly improving the accuracy of early warnings. The research findings provide valuable experience and reference for the monitoring and warning of high slopes in mountainous areas.

## 1. Introduction

Landslides have always been one of the deadliest geological disasters distributed worldwide, leading to the collapse of houses and fatalities [1,2,3,4,5]. The primary driving force behind landslide disasters is the gravitational force acting on rock and soil masses, making landslides most prevalent in mountainous areas. Specifically, geological structures such as discontinuities and properties, rainfall, groundwater seepage, earthquakes, and other factors often play a key role in causing slope instability.

The investigation into the influential mechanisms of stability factors has become the most crucial approach to distinguish the development stage and failure mode of landslides. Generally, the triggering factors of a landslide are multiple [6,7]. Among these factors water is one of the most frequent causes of landslides. Landslides triggered by water can be categorized into two types: water seepage inside the soil mass and surface flow. The primary source of water is rainfall. When rainfall carries a substantial amount of rainwater that seeps into the receiving soil, it results in a decrease in soil suction and an increase in soil pore water pressure. This, in turn, decreases soil resistance and ultimately leads to slope failure [8,9,10]. Consequently, many landslides are caused by rainfall [11,12]. The correlation between surface water flow and landslides lies in the erosion and damage to the slope surface, which further impacts the rock and soil structure of the slope, resulting in terrain and landforms conducive to instability [13,14].

As the soil mechanical properties decrease with surface water infiltration, various phenomena characterizing landslides occur, such as shear failure of rock and soil, groundwater outflow, surface fissures, and even the toppling of surface vegetation in the initial and evolution stages of landslides [15,16,17,18,19]. By monitoring these characteristics, the development stage of landslides can be predicted, and corresponding disposal measures can be taken to prevent further slope collapse [20,21]. Landslide prevention methods include brushing and reducing the load, gentle slope treatment, drainage, and backpressure at the foot of the slope, strengthening the foot of the slope, and reinforcing the slope body [22,23,24,25,26]. Drainage measures are particularly crucial for rainfall-induced landslides, including surface drainage and groundwater drainage. Therefore, drainage channels and pipes are commonly employed methods. Others include prestressed staggered anchor cables, retaining walls, anti-skid piles, pressure grouting, shotcrete, and grouting for seepage prevention. The monitoring of slope reinforcement measures includes surface deformation monitoring and internal monitoring. Surface deformation monitoring can be conducted through remote monitoring [27]. However, the variation of internal characteristics, such as deep displacement, groundwater pressure, and the stress and strain of piles or anchors, should be monitored using sensors [28,29]. In remote mountainous areas, the conditions for continuous on-site measurements are challenging, making it difficult to ensure the quality and consistency of monitoring data [30]. Additionally, conducting monitoring at multiple sites exacerbates the difficulties associated with on-site surveillance. Presently, the automation of landslide monitoring in remote mountainous regions remains a challenging task that is not easily accomplished.

In this study, we reported a landslide that happened in Yongshun County, Hunan Province in China. Through the collection of various surface features characterizing landslides, the destructive patterns and triggering mechanisms of slope landslides were analyzed. Based on this analysis, the stages of slope landslide occurrences were determined, and corresponding reinforcement and remediation measures were proposed. An intelligent monitoring and early warning system applicable to remote mountainous areas was developed. This system, based on Internet of Things (IoT) technology, integrated and developed a local IoT for key project monitoring devices such as surface displacement, deep deformation, groundwater level, and internal forces in anchored structures. The research findings can serve as an experiential reference for the monitoring and early warning of high slopes in mountainous areas.

## 2. General Characteristics of the Landslide

**Figure 1:**
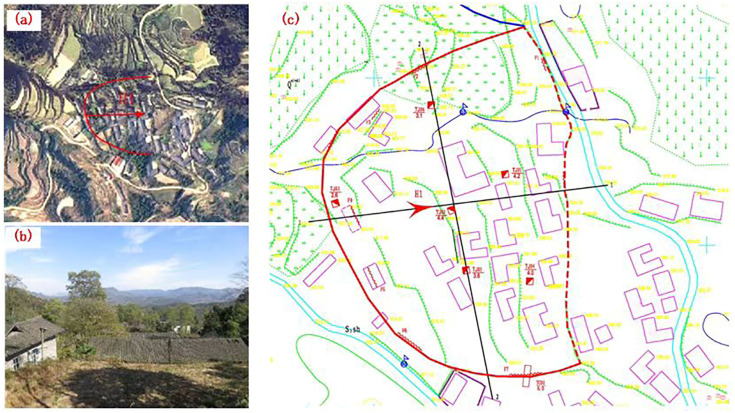
Distribution of landslide and the threatened area: (**a**) remote sensing image, (**b**) threatened building, (**c**) topographic of the landslide area.

The Xianeyu landslide, situated in Aimin Village, Yongshun County in Hunan Province of China (109°59′12″ E, Latitude: 29°15′22″ N), represents a considerable geological hazard with an estimated volume of approximately 10.5 × 104 m^3^, as shown in Figure 1. Once the landslide slides down, it directly threatens the life and property safety of over 220 people. This landslide was initially documented in June 1984, triggered by persistent heavy rainfall, leading to deformation of the landslide mass and the development of cracks in houses situated at its rear edge. In the 2003 monsoon season, heavy rainfall caused the displacement of approximately 3500 m^3^ of soil and rocks, resulting in damage to 12 houses.

In recent years, the Xianeyu landslide has exhibited more pronounced deformation, aggravated by the increasing frequency of extreme weather events. The mass of the landslide has significantly subsided, causing a noticeable tilt in wooden structures on its slope, localized shear cracks at the front edge of the landslide, and multiple instances of dry-stone retaining walls collapsing. Additionally, to the north of this landslide, there is an unstable sloped area. Field observations have indicated gradual subsidence in the cultivated fields on the slope, although it does not pose an immediate threat to structures. Therefore, it is recommended to enhance monitoring efforts for this unstable slope.

### 2.1. Geological Characteristics

Based on surface deformation characteristics and micro-topographic features, the rear and front edges of the landslide are demarcated by steep slopes, while the lateral boundaries of the landslide are determined by the presence of fissures and the ridge line. The plan view of the landslide exhibits a round-chair shape, and its longitudinal profile appears as a broken-line pattern with the main sliding direction oriented at 85°. The elevation of the landslide’s rear edge ranges from approximately 616.75 to 627.52 m, while the front edge’s elevation is between 579.59 and 583.78 m, resulting in a relative height difference of about 40 m. The landslide has an inclined length of approximately 142 m and a lateral width of about 210 m, covering an area of approximately 23,600 square meters. The thickness of the landslide ranges from 3 to 5 m, with an estimated volume of approximately 105,000 cubic meters. It is classified as a medium-sized shallow translational landslide composed of soil material.

**Figure 2:**
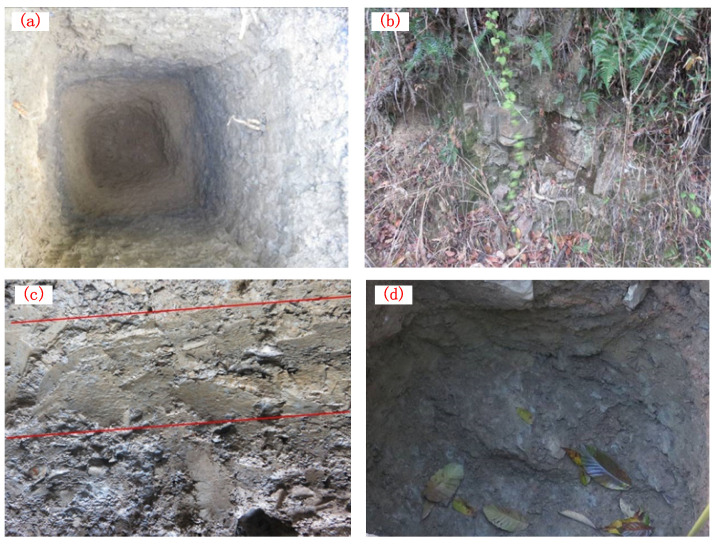
(**a**) Exploratory pit, (**b**) Quaternary residual layer, (**c**) the Upper Silurian Gauze Hat Group, (**d**) the Middle Silurian Luojiaping Group.

According to the on-site survey results as shown in Figure 2, the predominant geological strata in the affected area consist of Quaternary residual slope deposits (Q), as depicted in Figure 2b, which include fine-grained clay and gravelly soil. Additionally, there are Silurian upper Shamao Group (S3sh, Figure 2c) formations characterized by gray-green, gray–white, and purple–red mudstone, sandy shale, and quartz sandstone. The Silurian middle Luoreping Group (S2Lr, Figure 2d) is also presented as siltstone and sandstone. The geomorphological type of the landslide area falls under the category of low mountain fold-valley terrain, with an overall west-high and east-low topography (Figure 2). The composition of the landslide’s soil includes gravelly clay and soil forming a single-layer structure with relatively loose characteristics. The rock formations in the area range from weak to moderately hard and vary in thickness including laminated clastic rocks such as shale, slab-like shale, sandy shale, and mudstone. The rocks exhibit a strongly weathered condition, and there is extensive development of joint fractures within the rock layers, resulting in considerable fragmentation. Consequently, the rock strata exhibit relatively low resistance to deformation.

### 2.2. Weather Conditions

The study area experiences a humid mid-subtropical monsoon climate, characterized by an annual average temperature of 16.5 °C and precipitation totaling 1344.6 mm per year. However, since autumn 2009, the area has been facing an unprecedented series of droughts, causing extensive cracking in both the bedrock and soil. Meteorological data from the Yongshun County weather station reveal an average of 164.0 rainy days annually, with the longest consecutive rainy period lasting 20 days in May 2005. The annual rainfall is concentrated from April to September, accounting for 75% of the total annual rainfall. The average annual rainfall is 1344.6 mm. A 50-year maximum rainfall record is 95.8 mm/h. The highest annual rainfall recorded was 1837.7 mm in 1995, with a peak daily rainfall of 344.1 mm on 31 May of the same year. There are noticeable variations in rainfall distribution across Yongshun County, with higher rainfall occurring along the Longjiazhai syncline axis and the mountain township line in the north and west, while lower rainfall is observed in the south and east regions. Three times of extreme weather events were recorded, 167.0 mm rainfall on 23 July in 1993, 344.1 mm rainfall on 31 May in 1995. and 235.2 mm rainfall on 9 July in 2003. These precipitation patterns significantly impact soil saturation and water infiltration, which contribute to landslide formation. Over time, continuous rainfall infiltration has gradually exacerbated slope deformation, leading to the hazardous conditions observed today.

### 2.3. Deformation Development Characteristics

**Figure 3:**
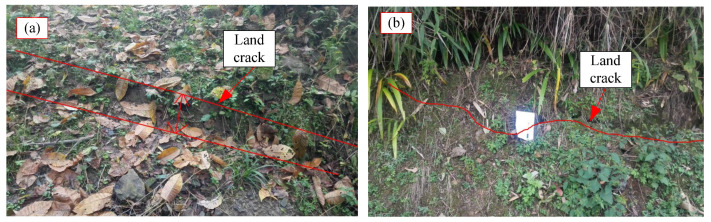
Cracks induced by landslide: (**a**) Cracks in rear edge of landslide, (**b**) Cracks in front edge of landslide.

**Figure 4:**
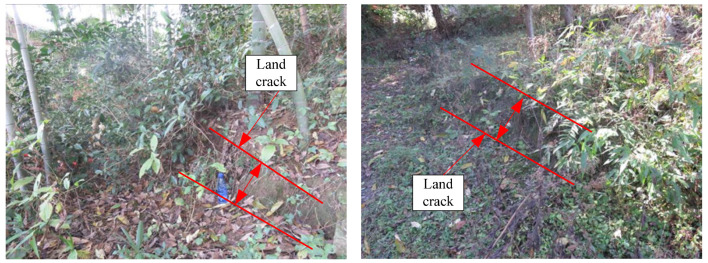
Cracks in side edge of landslide.

**Figure 5:**
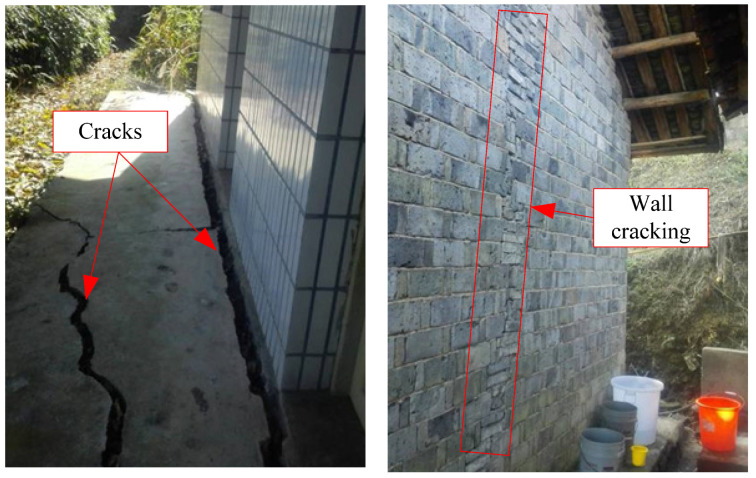
Cracks on wall and ground.

**Figure 6:**
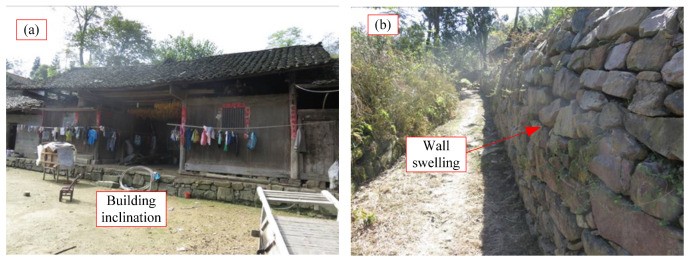
Phenomenon in front of landslide, (**a**) Building inclination, (**b**) Wall swelling.

As shown in Figure 3, Figure 4, Figure 5 and Figure 6, numerous cracks of varying sizes are now visible in the site located at the rear of the landslide. These cracks typically measure between 1 and 5 cm in width and 3 and 10 m in length. Among them, the most severe crack spans about 5 m in length and 0.5 m in width, causing noticeable leaning in the central part of the housing affected by the landslide. Additionally, tensile cracks emerged at the rear and sides of the landslide, ranging from 5 to 20 m in length, 0.1 to 0.2 m in width, and with displacement distances ranging from 0.1 to 0.3 m. At the front, shear cracks have become apparent, leading to the bulging and collapse of several dry-stone walls. These cracks are distributed around the landslide and form the sliding range on the surface after coalescence. At the top of the landslide, tensile cracks are the main type (Figure 3), while tensile shear cracks are more common on both sides of the landslide body (Figure 4). At the front edge of the landslide body, soil swelling cracks are the main type (Figure 6). And obvious inclination could be seen in the buildings on the landslide (Figure 5). All these phenomena indicate that the landslide was in a sliding stage. When cracks rapidly expand, it indicates that the landslide is accelerating its sliding.

The signs of deformation on the landslide mass are quite prominent, indicating that it is currently experiencing overall creep deformation. Tension cracks have emerged at the rear and sides of the landslide, with noticeable cracks appearing in houses situated at the rear of the landslide, as well as numerous dry-stone walls along the slope and at the front showing signs of bulging and collapsing. Local monitoring personnel have reported that the width of ground and house cracks is progressively increasing year by year. These observations strongly suggest that the Xianeyu landslide in Aimin Village is facing increased downslope force and reduced resistance, thereby further compromising its stability, especially during heavy rainfall or rainy conditions. Looking ahead, considering the influence of rainfall and human engineering activities, the trend of landslide deformation and rupture is expected to escalate, heightening the risk of landslide movement in the future.

## 3. Failure Mechanism of the Landslide

The mechanism of landslide destruction is influenced by a combination of internal and external factors. Internal factors encompass geological elements such as terrain, lithology, and structure, whereas external factors consist of continuous heavy rainfall and human activities.

In the specific area under consideration, the landscape features a karst hilly terrain with a moderately dissected topography and ground slopes typically ranging from 25° to 40°, providing favorable terrain conditions for landslide occurrence. The landslide mass primarily consists of residual soil mixed with a substantial amount of gravel. This soil type is characterized by its loose structure, mixed particle sizes, good permeability, and relatively low cohesion, rendering the slope vulnerable to instability after rainfall. Such susceptibility to rainfall-induced instability is considered one of the primary internal factors contributing to landslides in the area.

Additionally, meteorological factors play a crucial role in influencing landslide stability. In this region, the frequent occurrence of heavy rainfall, combined with the relatively large pores in the landslide mass soil, facilitates rapid infiltration of rainwater into the slope. This influx increases both the self-weight and downward force of the slope. Furthermore, intense rainfall can create hydraulic flow paths within the soil, leading to softening and saturation of the soil near these paths. Consequently, the soil’s shear strength decreases, diminishing its internal friction and cohesion, thereby heightening the risk of slope instability and sliding. Moreover, the presence of numerous houses within the landslide mass introduces additional load to the slope. Moreover, the steep slopes formed by road construction and other activities at the front edge of the landslide serve as significant contributing factors to landslide formation.

Based on investigations and field surveys, the primary deformation characteristics of the landslide mass manifest as cracks at the rear (Figure 3), sides (Figure 4), and bulging (Figure 6) at the front edge. The mechanism behind this formation primarily includes several factors: unstable slopes formed due to human-induced slope cutting at the front, the loading of residential houses at the rear and on the landslide mass, saturation of the soil due to rainfall and water infiltration, and increased weight of the landslide mass resulting in decreased shear strength, leading to gradual creeping towards the free edge under its own weight. This process exerts pressure on the soil at the front edge, causing it to press against the dry-stone retaining walls and resulting in their bulging. Additionally, the soil at the rear edge of the landslide mass, under the influence of gravity and artificial structures, pushes against the soil at the front edge, resulting in tension cracks at the rear and partial cracking at the front, thereby forming potential geological hazard points.

Further observations indicate that the deformation and destruction mode of the landslide belongs to the translational type. The deformation characteristics generally involve rapid shear sliding of the landslide towards the free edge under the pushing of soil mass at the rear edge while the shear surface is controlled by weak structural planes. The deformation progresses gradually from deep potential shear surfaces to the surface, with the rear edge of the landslide and the shear exit located at the terrain change turning point. Therefore, the deformation and destruction mode of the landslide belongs to the translational type.

## 4. Reinforcement Measures

The design of reinforcement engineering should adhere to the principles of safety, rationality, and economy. The focus should be on ensuring convenience in construction, minimizing environmental impact, and adopting the most cost-effective structural form while guaranteeing safety and normal functionality [31]. Moreover, it is crucial to align these principles with the goal of protecting the local ecological environment. Considering that the landslide mass in this area is primarily composed of soil and that there are numerous buildings and crops within the sliding range, the slope stabilization must ensure that the displacement of the slope is effectively controlled after reinforcement. The stabilization measures should be systematically arranged, with minimal land occupation. Therefore, a comprehensive stabilization approach combining anti-slide piles, retaining walls, and drainage (interception) systems is adopted. Comparatively, if anchor rods or anchor cables were used, the required land area would be larger, and the slope could still experience displacement after reinforcement, which would not adequately ensure the safety of the buildings above. This integrated approach ensures a balanced strategy that effectively mitigates landslide risks while also considering environmental conservation efforts.

**Figure 7:**
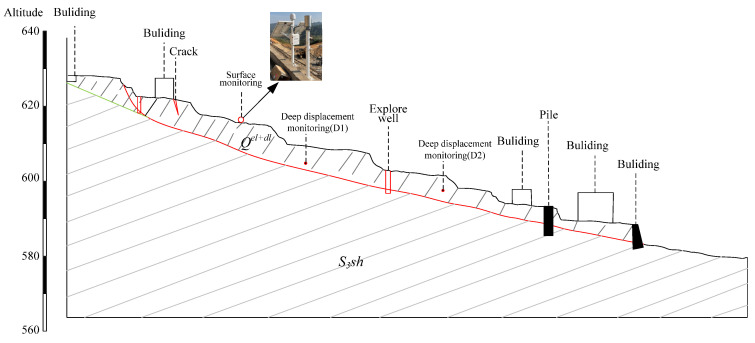
Site treatment of the landslide.

Figure 7 illustrates the plan for slope retaining treatment. Given the extensive length and significant pushing force of the landslide mass, anti-sliding piles are chosen for anti-sliding support. Considering the dense housing at the rear edge of the landslide mass and the limited construction space, anti-sliding pile engineering is implemented at the front edge of the landslide mass to provide necessary support against sliding. The anti-slide piles are deeply embedded and arranged to pass through the sliding surface, effectively preventing further movement of the slope soil mass. Moreover, the anti-slide piles can efficiently control the local deformation and displacement of the landslide and transmit the stresses generated by sliding to the more stable deep soil or bedrock. By using anti-slide piles in this scenario, the sliding forces from the upper sliding body can be resisted, achieving the effect of controlling the slope movement.

Retaining walls are predominantly positioned at the front edge of the landslide mass. Due to steep slopes resulting from road construction or house cutting, soil erosion and local bulging are common occurrences. Hence, retaining walls are strategically designed to be erected at the front of these steep slopes to stabilize the landslide mass. The retaining wall is installed at the front edge of the landslide mass to prevent further sliding of the landslide body. In combination with anti-slide piles, this creates a slope retaining system that ensures the safety of the structures located above.

Furthermore, cut-off and drainage ditches are strategically placed around the landslide to redirect seasonal surface water from atmospheric rainfall and domestic water usage by villagers into nearby valleys. Drainage ditches are constructed to facilitate the efficient discharge of surface water within the landslide area during the rainy season. This measure effectively reduces soil erosion and minimizes rainfall infiltration within the landslide area, which may contribute to enhanced stability.

## 5. Monitoring

### 5.1. Optimization and Deployment of Monitoring Systems

To effectively monitor landslides, it is crucial to conduct safety risk identification through typical high-risk slope assessment. This involves employing various monitoring terminals to achieve comprehensive real-time online monitoring of slope displacement, rainfall, surface water, groundwater, stress, macro deformation, and other relevant factors. These monitoring tools enable continuous and real-time safety monitoring of high-risk slopes, ensuring timely detection and response to potential landslide hazards. The principle of IoT monitoring technology involves collecting physical data through sensors, transmitting the data via wireless communication technologies, and processing it in real time using edge computing or cloud computing. This is combined with intelligent analysis and decision-making models to provide scientific support for the management and control of monitored objects. This technology is widely applied in various fields, including industrial equipment monitoring, environmental monitoring, intelligent transportation, and building safety monitoring, significantly enhancing monitoring efficiency and accuracy.

In addition to the traditional monitoring system, on-site network communication among various devices using LoRa wireless self-organizing network technology is explored. This enables devices to function independently of remote servers, allowing the collection, calculation, and transmission of Beidou monitoring data directly from high-risk slopes at the project site. This approach reduces overall data transmission requirements, lowers the system’s demands for on-site communication environments, and unlocks cloud computing capabilities. It fosters integrated collaborative operation of terminal edge management cloud network, thereby enhancing the system’s adaptability and intelligence. Moreover, an integrated approach is implemented, along with high-performance batteries and solar panels, to create a self-powered Internet of Things composite sensor monitoring unit.

Specifically, for the on-site self-organizing network scheme, the LoRa gateway incorporates a digital baseband chip based on SX1301, featuring an eight-channel single gateway capable of accommodating 300 terminals, with a total power consumption of approximately 6 W. It boasts a coverage area of up to a 3 km radius in an open environment, offering superior anti-interference and link stability compared to FSK technology. By integrating a 160 W monocrystalline solar panel, a 120 AH lead-acid gel battery, a solar controller, and other self-built photovoltaic power supply systems, we achieve 24-h power supply with uninterrupted power for 15 consecutive rainy days. The LoRa nodes utilize the LoRaWAN protocol with DSSS modulation and operate in the half-duplex communication mode. Each node has a built-in microcontroller with an integrated transceiver program, supporting common baud rates ranging from 1200 to 57,600. The gateway based on the SX1301 chip adopts an eight-channel parallel data transmission and reception scheme. This approach significantly improves data throughput compared to traditional LoRa polling methods while also reducing data congestion and minimizing transmission conflicts.

The composite sensor monitoring units communicate using the IEEE802.15.4 standard signal [32], with a transmission range of 50–100 m, enabling low-power self-organizing networks. For each composite sensor monitoring unit or a group of adjacent units, an intelligent data acquisition system is developed. This system can simultaneously collect data from various sensors, including GNSS, fixed inclinometers, rain gauges, anchor rod load cells, anchor cable load cells, piezometers, strain gauges for lattice structures, retaining wall earth pressure cells, and more. The collected data are then integrated and transmitted at predefined data transmission intervals.

**Figure 8:**
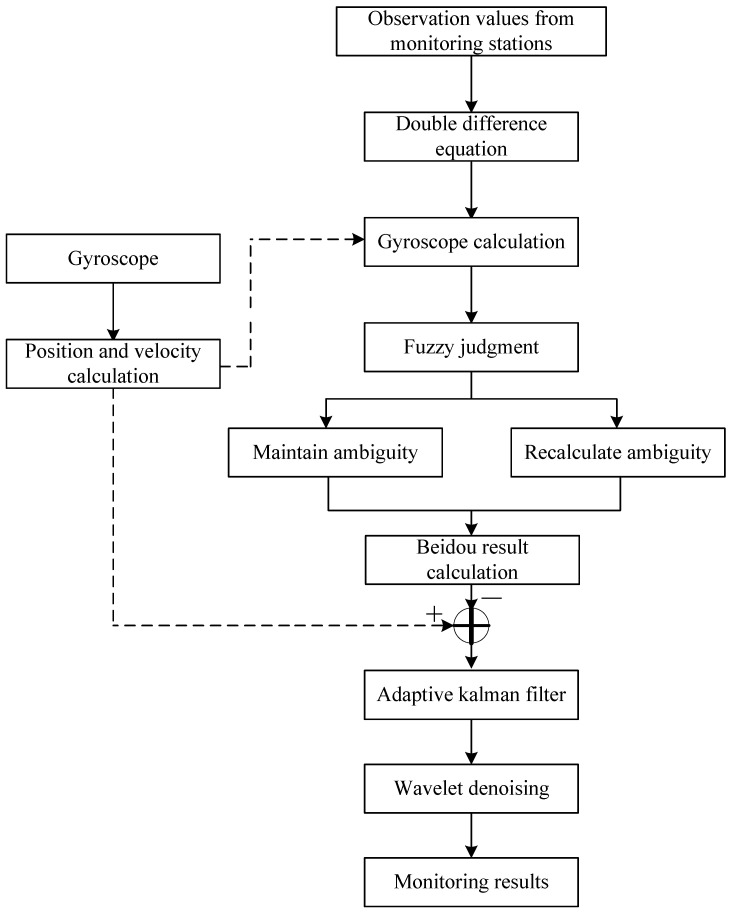
Algorithm for gyroscope fusion.

The fusion calculation technology of Beidou and gyroscope is adopted (Figure 8), involving the integration of a gyroscope device into the low-power Beidou device. The monitoring data from both devices are fused, and the gyroscope monitoring results are incorporated as constraints when fixing the ambiguity of the Beidou solution algorithm. Additionally, adaptive Kalman filtering and wavelet denoising processing are applied to the monitoring results of both devices to yield stable monitoring outcomes. This approach effectively addresses challenges such as ambiguity fixation difficulties, low accuracy, and instability in monitoring.

Considering the large volume of real-time monitoring data and the communication challenges in mountainous high-slope areas, especially during heavy rainfall when the probability of landslides significantly increases, it is a critical moment for slope monitoring and early warning. Therefore, localized deployment of data acquisition and processing systems is necessary, providing on-site computation capabilities. This approach enables data collection, real-time computation, post-processing, and transmission of results based on LoRa IoT networks. By shortening the data acquisition chain and improving reliability, this method enhances the efficiency of monitoring and early warning systems.

### 5.2. Results

As shown in Figure 7, surface and deep-seated displacement monitoring is consistently conducted on the slope to facilitate long-term real-time monitoring and early warning. Furthermore, recognizing the significant correlation between rainfall and landslides in this area, rainfall gauges are strategically installed.

Since the initiation of the site treatment project in 2022, the on-site monitoring instruments have been sequentially deployed and put into operation. Consequently, the monitoring system has been operating continuously and reliably. Data forecasting adheres to a daily reporting strategy and is accessible in real time via the internet cloud platform. In the event that monitored data surpass preset threshold values, the system automatically issues alerts and delivers varying levels of warning messages based on the magnitude of the monitored values. Moreover, it provides on-site disposal recommendations.

**Figure 9:**
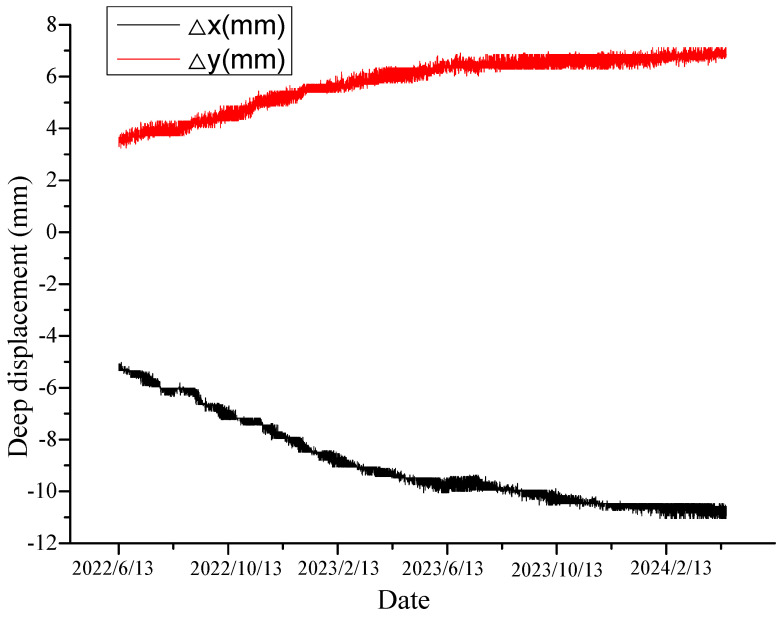
Deep displacement.

**Figure 10:**
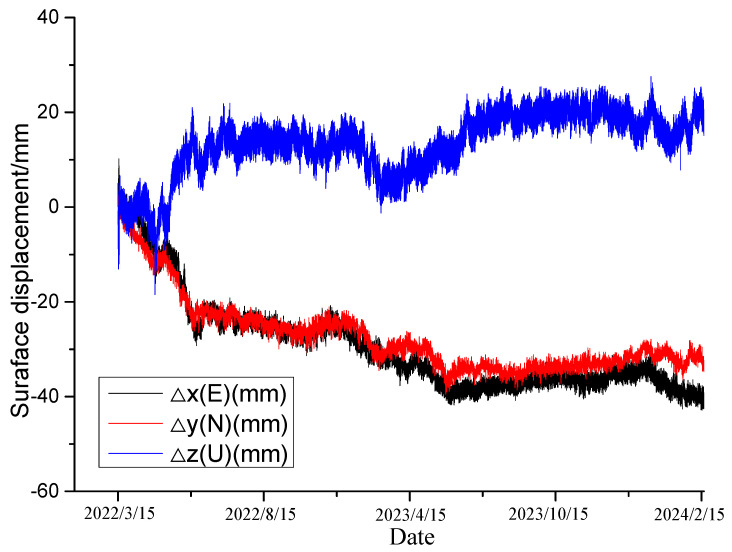
Surface displacement.

**Figure 11:**
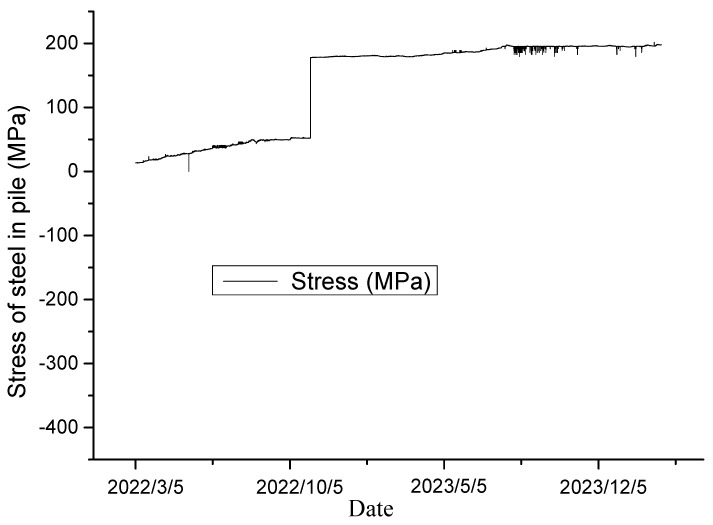
Stress of steel in pile.

**Figure 12:**
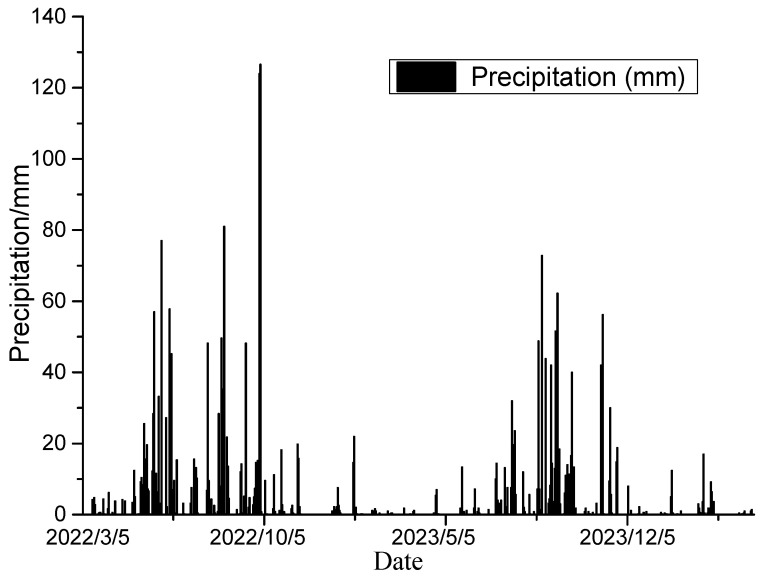
Monitored precipitation.

Figure 9, Figure 10, Figure 11 and Figure 12 exhibit the monitoring and forecasting data of select monitoring instruments from the commencement of their operation until the present. An examination of these figures reveals that since the slope’s construction, surface displacement, deep-seated deformation, and stress of the pile reinforcement have exhibited gradual increases in the initial stage, followed by stabilization over time.

As shown in Figure 9, illustrating the deep-seated displacement–time curve, and Figure 10, showing the surface displacement curve, both the surface and deep-seated displacements of the rock–soil mass have continuously increased over time following the disturbance caused by excavation, gradually stabilizing afterward. This suggests that the deformation of the deep-seated rock–soil mass was effectively controlled following the completion of the supporting structure. At this juncture, all reinforcement facilities of the slope amalgamated into an organic whole, successfully regulating the displacement rate of the slope. The change pattern of the monitoring system data can accurately reflect the real-time working status of each reinforcement facility. The monitoring results of the stress monitoring (Figure 11) indicate that it began to exert the most significant effect after the completion of the anti-sliding pile. A considerable change in stress was observed in early November 2023, followed by gradual stabilization. This suggests a reduction in slope deformation under the support of the anti-sliding piles and approaching a stable state.

The rainfall monitoring results depicted in Figure 12 automatically trigger warning messages when the rainfall exceeds the predefined threshold value within a short period. The threshold values for daily rainfall are set as follows: 55 mm for a blue alert, 60 mm for a yellow alert, 80 mm for an orange alert, and 100 mm for a red alert. Since its establishment, the system has issued warnings 13 times, each corresponding to rainfall exceeding 55 mm. Following the issuance of the warning, the system automatically provides recommendations for on-site observation of the slope status, along with continuous warning alerts. On-site observations reveal that the slope boasts a well-functioning drainage system. Although rainfall does have some influence on surface displacement, it has not reached a level that threatens the overall stability of the slope.

## 6. Conclusions

The performance of a case study on a landslide that occurred in Yongshun County, Hunan Province, China was reported. The investigation involved geological surveys of the landslide, analysis of regional climatic characteristics, and historical analysis of landslide development to understand its mechanism. The findings indicate that the landslide in this area is a shallow translational soil landslide primarily caused by long-term creep influenced by rainwater infiltration. To address the layout of houses, topography, and characteristics of the landslide within the area, reinforcement measures including the use of anti-sliding piles and retaining walls were implemented. Recognizing the influence of environmental factors on monitoring signals in remote mountainous areas, low-power, low-cost, high-precision Beidou receiving devices were developed based on existing Beidou monitoring equipment. The application of LoRa self-organizing network technology in the intelligent transformation of the monitoring system enabled the integration of end edge management cloud network operations, enhancing the adaptability and intelligence of the system and addressing the issue of decentralized data in slope engineering monitoring. Utilizing a fusion of Beidou and gyroscope-based calculation techniques for local preliminary data processing, the integration and upgrade of monitoring data were achieved, enabling data collection, real-time calculation, post-processing, and transmission of calculation results based on LoRa IoT technology. This streamlined data collection process enhances the reliability of deformation monitoring and early warning for typical high slopes in mountainous regions, demonstrating reliable results and significant potential for widespread application.

## Data Availability

All data used in the study was shown in the manuscript.
